# Rhinocerebral mucormycosis and *Trichosporon asahii* fungemia in a pediatric patient with acute lymphoblastic leukemia: a rare coinfection

**DOI:** 10.1590/S1678-9946202466041

**Published:** 2024-07-08

**Authors:** Liuyang Hu, Guiliang Liu, Xingchun Chen

**Affiliations:** 1 Guangxi Academy of Medical Sciences, The People’s Hospital of Guangxi Zhuang Autonomous Region, Department of Laboratory Medicine, Nanning, Guangxi, China; 2 Guangxi Academy of Medical Sciences, The People’s Hospital of Guangxi Zhuang Autonomous Region, Department of Pediatrics, Nanning, Guangxi, China

**Keywords:** Rhinocerebral mucormycosis, Trichosporon asahii, Coinfection, Acute lymphoblastic leukemia

## Abstract

Mucormycosis is a rare life-threatening opportunistic infection, with rhinocerebral mucormycosis (ROCM) being the most common presentation. *Trichosporon asahii* is an emerging pathogen that often causes fatal infections in patients with underlying hematologic malignancies due to its high drug resistance. We report a rare case of concomitant rhinocerebral mucormycosis and *T. asahii* fungemia secondary to *Pseudomonas aeruginosa* sepsis in a patient with neutropenia and acute lymphoblastic leukemia. A boy aged one year and two months was diagnosed with B-cell acute lymphoblastic leukemia on January 10 and underwent three courses of regular chemotherapy. He experienced neutropenia for 154 days and was hospitalized for vomiting, diarrhea and fever for 3 days. The day after hospitalization, *Pseudomonas aeruginosa* was isolated by blood culture and ceftazidime/avibactam was administered. Extracorporeal Membrane Oxygenation (ECMO) was used to provide continuous extracorporeal respiration and circulation for the patient. On day 8, the patient developed *T. asahii* fungemia. On day 10, he presented with necrotizing skin caused by *Rhizopus delemar*. He was treated with liposomal amphotericin B for *Rhizopus delemar* and voriconazole for *T. asahii* infection. Unfortunately, his health deteriorated and he died on day 11 due to the rapid progression of the infection and multiple organ failure. The management and treatment of such a complex infection requires a multidisciplinary approach and close monitoring of the patient’s condition. Therefore, it is imperative to continue to research and report rare cases such as this to further understand the complexities of mucormycosis and trichosporidiosis coinfection and improve patient outcomes.

## INTRODUCTION

Mucormycosis (also known as zygomycosis) is a rare life-threatening opportunistic infection, with rhinocerebral mucormycosis (ROCM) being the most common presentation^1^. It is an infection with aseptate molds of the order Mucorales. Despite the name, *Mucor* spp. are not the only causative agents of mucormycosis, as fungi of the genera *Rhizopus, Saksenaea, Cunninghamella, Apophysomyces, Lichtheimia, Cokeromyces* and *Rhizomucor* can also be involved^2^. *Rhizopus* is the most common causative agent of mucormycosis, accounting for approximately 60% of reported cases^2^. *Trichosporon asahii* is an emerging pathogen that often causes fatal infections in patients with underlying hematologic malignancies due to its high drug resistance^3^. *T. asahii* is the most common species isolated from invasive trichosporidiosis, and approximately 80% of cases are secondary to fungemia, resulting in a high mortality rate (40-90%)^3^. The major risk factors for mucormycosis and trichosporidiosis are immunocompromised hosts, hematologic malignancies, neutropenia, solid organ transplantation, autoimmune diseases, multiple injuries, central venous catheters, corticoid use and surgery^3,4^. Accurate identification of *Mucorales* species and *T. asahii* is essential to initiate pathogen-specific therapy, due to the high resistance of these species to certain antifungal drugs. We report a rare case of *T. asahii* fungemia associated with ROCM secondary to *Pseudomonas aeruginosa* sepsis in a patient with neutropenia and acute lymphoblastic leukemia.

## CASE REPORT

**Figure 1:**
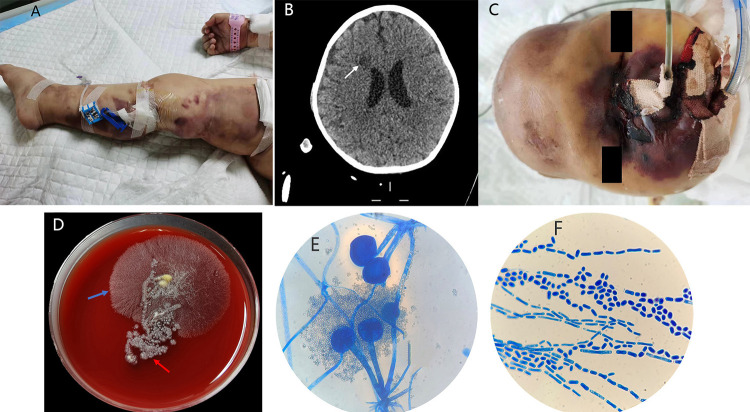
A) Symmetrical large purpura on the patient’s limbs; B) Computed tomography of the brain showing multiple infarcts in the right frontal lobe (white arrow); C) Necrotic lesion rapidly expanding to the entire face; D) Necrotic tissue culture yielding two types of fungi on blood agar, the blue arrow indicates *Rhizopus delemar* and the red arrow points to *T. asahii*; E) *Rhizopus delemar* lactophenol cotton blue stain showing stolons and pigmented rhizoids and the formation of sporangiophores, singly or in groups, from nodes directly above the rhizoids, x1000;F) Budding spores and arthrospores on *T. asahii* lactophenol cotton blue stain, x1000.

A boy aged one year and two months was diagnosed with B-cell acute lymphoblastic leukemia on January 10, 2022. He underwent three courses of regular chemotherapy and experienced neutropenia for 154 days. On August 17, 2022, he was admitted to the Department of Pediatrics of the People’s Hospital of Guangxi Zhuang Autonomous Region (Nanning, China) due to vomiting, diarrhea and fever for 3 days. Physical examination revealed that he had a fever (39.2 °C), tachycardia (180 beats/min) and hypotension. His partial pressure of oxygen was 114 mmHg (oxygen concentration 29%) and his oxygen saturation was 99.4%. Abnormal laboratory findings included pancytopenia (leukopenia [0.48×10^9^/L, normal values 5.1~14.1×10^9^/L], anemia [hemoglobin: 73g/L, normal values 107~141g/L] and thrombocytopenia [41×10^9^/L, normal values 190~524×10^9^/L]), impaired liver function (ALT 154U/L, normal values 7~40U/L; AST 102U/L, normal values 13~35U/L ), hypernatremia (Na+165mmol/L, normal values 135~145mmol/L), high C-reactive protein (149.63mg/L, normal values 0~10 mg/L), high procalcitonin (>100.00ng/mL, normal values 0~0.05 ng/mL ) and high IL-6 (>5000.00pg/mL, normal values 0~7pg/mL). The patient was initially diagnosed with sepsis, and meropenem was used for antibacterial therapy without a clear infectious etiology after inter-consultation with the infectious disease team. In addition, the patient received supportive circulation, high-dose vasoactive drugs and multiple transfusions of platelets, cryoprecipitate, fresh frozen plasma and granulocyte colony-stimulating factor (G-CSF). On the day after hospital admission, ECMO was used to provide continuous extracorporeal respiration and circulation for the patient. *Pseudomonas aeruginosa* (susceptible to cefperazone/sulbactam, piperacillin/tazobactam and ceftazidime/avibactam) was isolated via blood culture, and ceftazidime/avibactam was administered for antibacterial therapy. However, there was no significant clinical improvement. On the 3rd day of hospitalization, it was found that the patient had symmetric large purpura gradually spreading to his limbs and trunk (Figure 1A). On the 4th day of hospitalization, the patient received bedside continuous renal replacement therapy (CRRT) for hypernatremia and anuria. On the 7th day of hospitalization, he developed worsening jaundice and his liver was significantly enlarged to 9 cm below the rib.

On the 8th day of hospitalization, direct microscopic examination of positive blood culture showed fungal spores, and antifungal treatment with caspofungin was started. On the 9th day of hospitalization, the patient developed skin necrosis on the right nasal wing and eye corner, with unequal pupils (2 mm on the left and 5 mm on the right) and dull response to light on the left side. These findings did not rule out intracranial hemorrhage, but strongly suggested that the skin necrosis near the right eye had damaged the nerves and muscles that control the pupil. Further laboratory tests, including a CT scan, revealed multiple infarcts in the right frontal lobe of the patient’s brain (Figure 1B). In addition, 1-3-β-D-glucan showed an elevated level (188.86 pg/mL). *T. asahii* was further recovered by blood culture using MALDI-TOF MS, with a minimum inhibitory concentration (MIC) of 2 µg/mL for amphotericin B, 0.03 µg/mL for voriconazole, 0.5 µg/mL for itraconazole and 2 µg/mL for fluconazole. Thus, voriconazole was added for antifungal treatment. On the 10th day of hospitalization, necrosis rapidly spread to almost the entire face of the patient (Figure 1C). Necrotic tissue culture yielded two types of fungi on blood agar: a yeast-like fungus and a filamentous fungus. The yeast-like fungus was identified as *T. asahii* by MALDI-TOF MS, and the filamentous fungus was identified as *Rhizopus delemar* by internal transcribed spacer (ITS) sequencing (94.99% nucleotide identity). In Figure 1D, the blue arrow indicates *Rhizopus delemar* and the red arrow points to *T. asahii* on blood agar. *Rhizopus delemar* is characterized by the presence of stolons and pigmented rhizoids and by the formation of sporangiophores, singly or in groups, from nodes directly above the rhizoids on lactophenol cotton blue stain (Figure 1E). Budding spores and arthrospores were seen on *T. asahii* lactophenol cotton blue stain (Figure 1F). The minimum inhibitory concentrations (MICs) for *Rhizopus delemar* were 0.5 µg/mL for posaconazole and 1 µg/mL for amphotericin B. The patient’s condition was complicated by Rhizopus-associated ROCM and *T. asahii* fungemia coinfection. As a result, the anti-infective regimen had to be changed. The patient was treated with ceftazidime/avibactam for *Pseudomonas aeruginosa* sepsis, liposomal amphotericin B for *Rhizopus delemar* infection and voriconazole for *T. asahii* infection. However, on the 11th day, his condition continued to worsen, ultimately leading to rapidly progressive infection and multiple organ failure, which resulted in his death.

## DISCUSSION

In recent decades, the incidence of ROCM in patients with hematologic malignancies has increased, resulting in high mortality^5^. Although the exact pathogenesis of mucormycosis remains unclear, it is known to involve extensive vascular invasion^6^, leading to vascular thrombosis and subsequent tissue necrosis. Therefore, early clinical suspicion and accurate identification of mucormycosis are essential. Treatment approaches for nasal-brain and localized skin infections often involve a combination of medical management and active surgical intervention^7^. It is also crucial to treat and improve any underlying conditions, such as COVID-19^8^, to improve patient survival, correcting acidosis and hyperglycemia, restoring white blood cell count and adjusting the use of immunosuppressive agents^9^.

Because *Mucorales* are resistant to voriconazole and mucormycosis often mimics aspergillosis, fusaridiosis and other fungal infections, accurate diagnosis is essential for proper management. The reversed halo sign on chest computed tomography has been associated with mucormycosis in neutropenic patients, but is not specific enough to confirm the diagnosis^10^. Direct fungal microscopy, histopathology and biopsy cultures of skin lesions are crucial for diagnosis. Molecular approaches that may be extremely useful for early diagnosis are also being developed^7^.

The most common symptoms of ROCM are eyeball protrusion, ophthalmoplegia and decreased visual acuity, which are not specific^1^. Manifestations of the disease can range from localized sinus disease to extensive orbital and intracranial involvement, often associated with neurological deficits. The only specific symptom of ROCM described in the literature is necrotic eschar with blackened nasal mucosa. However, facial necrosis remains a late sign, occurring in only 2% of patients^1^. The prognosis for localized sinus lesions that have not spread beyond the sinuses and undergo surgical debridement is relatively good, with a mortality rate as low as 0.10%^1^. It has been reported that the orbital involvement rate of rhinocerebral mucormycosis ranges from 66% to 100%, leading to increased mortality, and the survival rate of patients with fungal encephalopathy is very low^4^.

Combined surgical and medical management has been associated with improved survival. Surgical debridement of necrotic tissue may allow for better penetration of antifungal agents, thereby improving outcomes. Unfortunately, surgical debridement is not feasible in patients with intracranial extension. Despite appropriate antifungal treatment, the mortality rate of patients unable to undergo surgery is high, indicating the need for early diagnosis and better treatment strategies^11^. Thus, surgical debridement may be considered in patients with malignant hematologic disease even in the presence of leukopenia and thrombocytopenia^12^. A multicenter study showed that liposomal amphotericin B had a significantly lower all-cause mortality rate compared with other treatment options. Delayed initiation of liposomal amphotericin B treatment was associated with higher mortality rates^13^. Isavuconazole has also been recognized as an effective alternative when liposomal amphotericin B is intolerable or ineffective^14^. Posaconazole has shown promising results as a salvage therapy, although it is not as effective as liposomal amphotericin B^15^.

Trichosporon species have emerged as important opportunistic fungal pathogens, with *T. asahii* being the leading cause of disseminated infections and leading to significant morbidity and mortality in patients with hematologic malignancy. Neutropenia and the presence of a central venous catheter are significant factors contributing to Trichosporon infections, and the optimal treatment for Trichosporon is still under investigation^16^. *T. asahii* has been isolated from blood in 75% of pediatric cases^3^. Invasive *T. asahii* has been commonly observed with cutaneous involvement with maculopapular or pustular lesions, sometimes necrotic, indicating trichosporonosis^3^. Clinicians should maintain a high index of suspicion for trichosporonosis in pediatric patients with hematologic malignancy who present with persistent fever and neutropenia, especially when echinocandins are administered for antifungal prophylaxis^3^. Trichosporon is known to be inherently resistant to echinocandins and to have poor susceptibility to polyene drugs. Breakthrough trichosporosis may occur in patients receiving empirical antifungal therapy with echinocandins^17^. In the past decade, amphotericin B and fluconazole have also been reported as antifungal agents with a high risk of breakthrough trichosporosis, including prophylactic coadministration of fluconazole plus amphotericin B^17^. A number of studies have shown that the recommended first-line therapy for *T. asahii* is voriconazole, which is likely to be superior to amphotericin B, fluconazole and itraconazole for treatment, but successful outcome depends largely on the underlying immune status of the host^18^. The mortality rate was over 80% in patients with hematologic malignancy, and survival is thought to be primarily related to bone marrow recovery. In the reported cases, cured patients did not have neutropenia at the time of diagnosis or recovered quickly from neutropenia^18^. Clinicians should maintain a high index of suspicion in patients with the aforementioned risk factors, especially those with hematologic malignancies who have catheters. Removal of the central venous catheter and treatment of the underlying immunosuppression may reduce mortality^18^.

According to our investigation, this is the third case of Mucormycosis and *T. asahii* coinfection. Table 1 shows the previously reported clinical characteristics of Mucormycosis and *T.asahii* coinfection^19,20^.

**Table 1:** Clinical characteristics of previously reported Mucormycosis and *T.asahii* coinfection.

Article	Year	Age (years)/sex	Symptoms	Primary lesion	Underlying conditions	Treatment	Outcome
De Decker *et al.* ^19^	2006	12/F	Skin and muscle necrosis	Leg necrotizing fasciitis	Traffic accident	Amphotericin B, Posaconazole, surgery, Hyperbaric oxygen	Survival
Ozkaya-Parlakay *et al*.^20^	2016	16/M	Cutaneous necrosis, nasal bone necrosis	Paranasal	Ewing sarcoma	Caspofungin, liposomal amphotericin B	Death
This report	2022	1/M	Skin necrosis	Right nasal wing and eye corner	Acute lymphoblastic leukemia	Amphotericin B, voriconazole	Death

## CONCLUSION

In this study, we presented a case of ROCM and *Trichosporon asahii* coinfection in an immunocompromised patient aged one year and two months. Acute lymphoblastic leukemia with neutropenia increased the risk of infection. In addition, the patient had an indwelling venous catheter, a medical device used for long-term intravenous treatment, which further increased the risk of developing coinfection. The management and treatment of such a complex infection requires a multidisciplinary approach and close monitoring of the patient’s condition. Patients with acute lymphoblastic leukemia and necrotic eschar should be closely monitored for *Mucorales* and *Trichosporon*. Reversal of risk factors, such as restoration of white blood cell count, adjustment of the use of immunosuppressive agents, early diagnosis, prompt and appropriate antifungal therapy and surgical debridement are essential for a favorable outcome.
